# Epigallocatechin Gallate-Modified Graphite Paste Electrode for Simultaneous Detection of Redox-Active Biomolecules

**DOI:** 10.3390/s18010023

**Published:** 2017-12-22

**Authors:** Hashwin V. S. Ganesh, Meissam Noroozifar, Kagan Kerman

**Affiliations:** 1 Department of Physical and Environmental Sciences, University of Toronto, Scarborough 1265 Military Trail, Toronto, ON M1C 1A4, Canada; hashwin.ganesh@mail.utoronto.ca; 2 Analytical Research Laboratory, Department of Chemistry, University of Sistan and Baluchestan, P.O. Box 98135-674, Zahedan, Iran; mnoroozifar@chem.usb.ac.ir

**Keywords:** modified graphite paste electrode, epigallocatechin gallate, green tea, simultaneous determination, real samples

## Abstract

In this study, simultaneous electrochemical detection of ascorbic acid (AA), dopamine (DA), and uric acid (UA) was performed using a modified graphite paste electrode (MGPE) with epigallocatechin gallate (EGCG) and green tea (GT) powder. It was shown that the anodic peak current increased in comparison with that of the graphite paste electrode (GPE) in the cyclic voltammograms. The optimal pH for simultaneous determination of a quaternary mixture of AA–DA–UA was determined to be pH 2. The anodic peak potentials for a mixture containing AA–DA–UA were well separated from each other. The catalytic peak currents obtained at the surface of the MGPE/EGCG were linearly dependent on the AA, DA, and UA concentrations up to 23, 14, and 14 µM, respectively. The detection limits for AA, DA, and UA were 190, 90, and 70 nM, respectively. The analytical performance of this sensor has been evaluated for simultaneous detection of AA, DA, and UA in real samples. Finally, a modified electrode was prepared using GT and used for simultaneous determination of AA, DA, and UA. Based on the results, MPGE/GT showed two oxidation peaks at 0.43 and 0.6 V for DA and UA, respectively, without any oxidation peak for AA. The calibration curves at the surface of MGPE/GT were linear up to 14 µM with a detection limit of 0.18 and 0.33 µM for DA and UA, respectively. MGPEs provide a promising platform for the future development of sensors for multiplexed electrochemical detection of clinically important analytes.

## 1. Introduction

Tea is one of the most widely consumed beverages in the world, with the main varieties being green and black. Green tea is produced by precluding the oxidation of polyphenols present in the leaf compared to black tea where oxidation of the polyphenols is promoted [1]. Green tea is rich in polyphenols such as flavanols and flavonols, which are predominantly found as catechins constituting up to 30% of dry leaf weight [2]. The major catechins found in green tea are catechin (C), (−)-epicatechin (EC), (−)-epicatechin 3-gallate (ECG), (−)-epigallocatechin (EGC), and (−)-epigallocatechin gallate (EGCG) [2].

**Figure 1:**
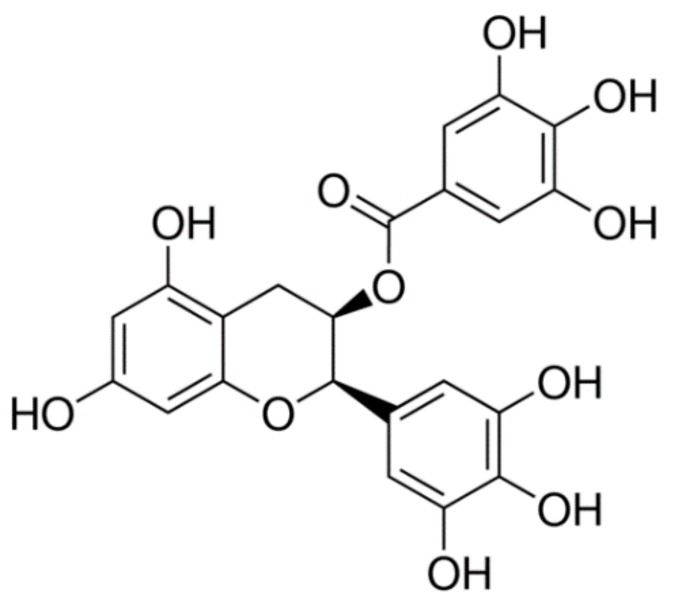
Structure of (−)-epigallocatechin gallate (EGCG).

EGCG, in particular, has been shown to have interesting properties including many potential therapeutic benefits such as anti-inflammatory [3,4], anti-cancer [5,6], and anti-steatotic [7] effects. The chemical structure of EGCG is shown in Figure 1. Research from our lab has also shown that EGCG interacts with key proteins implicated in Alzheimer’s disease (AD), such as amyloid-β [8], and also prevents aggregation of amyloid-β in the presence of metals such as copper [9]. In addition, EGCG has been reported to bind to amyloidogenic polypeptides, resulting in unstructured off-pathway oligomers, thereby shunting it away from AD-causing pathogenic pathways [10,11,12]. EGCG’s ameliorative effects are thought to depend on two main mechanisms: its ability to scavenge superoxide radicals generated during the reduction of metals (such as Fe) and its metal chelating ability [13]. 

Modification of electrode surfaces using organic molecules is an exciting area of research with many interesting applications. Electrochemical modification using oxygenated functional groups such as carboxylic, hydroxyl, quinone, and other ketonic groups have been reported [14,15]. In addition, electrochemical modifications based on electrochemical oxidation and reduction of functional groups such as alcohols, amines, carboxylates, and hydrazines have also been developed and studied [16,17,18,19]. Polyphenols are important organic molecules that have interesting chemical properties. Polyphenols contain one or more hydroxylated benzene rings and are differentiated by the position of the hydroxyl groups in their structure. Some of the polyphenols are electroactive, making them particularly attractive for electrochemical applications. In this study, we have developed an EGCG (green tea-derived polyphenol) modified graphite paste electrode (MGPE) for the simultaneous electrochemical detection of analytes such as ascorbic acid (AA), dopamine (DA), and uric acid (UA), using differential pulse voltammetry (DPV). In particular, we have exploited the reaction mechanism of the electrochemical oxidation of the phenol groups on EGCG to ketones for successful detection of the analytes.

Using the EGCG-modified graphite paste electrode (GPE), we were able to simultaneously detect AA, DA, and UA in the concentration ranges of 1–10 and 10–23 μM for AA, and 0.5–5 and 5–14 μM for both DA and UA. The theoretical detection limits were obtained as 190, 90, and 70 nM for AA, DA, and UA, respectively. 

Additionally, we have also reported a green tea (GT) powder modified GPE for the successful electrochemical detection of DA and UA using DPV. Since catechins constitute up to 30% of the dry leaf weight, we wanted to examine whether a GPE directly modified with green tea powder (Hamasaen, Japan) could be deployed for the detection of clinically important analytes. While the green tea powder-modified electrode was able to successfully detect two out of the three analytes (DA and UA), its performance was inferior compared to the EGCG–MGPE. This suggests that modifying electrodes with the pure extracted polyphenol compound, in fact, increases the performance of the electrochemical sensor.

## 2. Materials and Methods

### 2.1. Materials

Epigallocatechin gallate was purchased from Sigma-Aldrich (Oakville, ON, Canada). High purity graphite powder, ascorbic acid (AA), dopamine (DA), uric acid (UA), potassium ferrocyanide, and potassium ferricyanide were purchased from Sigma-Aldrich (Oakville, ON, Canada). Phosphate buffer solution (PBS) was prepared from H_3_PO_4_ (0.1 M); the pH range was adjusted to 2.0–5.0 with 0.1 M H_3_PO_4_ (Sigma-Aldrich, Canada) with NaOH (Sigma-Aldrich, Canada) solutions 0.1 M and these PBS solutions were used as supporting electrolytes. Green tea was purchased from Hamasaen (Japan). All solutions were prepared with ultra-pure Milli-Q water with a resistivity of 18.2 MΩ cm. 

### 2.2. Instrumentation

Electrochemical measurements and electrochemical impedance spectroscopy (EIS) were carried out with a µAutolab PGSTAT 128N (EcoChemie, Utrecht, The Netherlands) potentiostat/galvanostat controlled by NOVA 2.0 software. All electrochemical experiments were carried out in a conventional three-electrode cell at room temperature. A platinum counter electrode and an Ag/AgCl reference electrode were used in connection with a GPE and MGPE/EGCG or MGPE/GT as the working electrode. Electrochemical impedance spectroscopy (EIS) measurements were performed in 10 mM [Fe(CN)_6_]^3−/4−^ prepared in 0.1 M KCl over a frequency range of 0.1 Hz to 10 kHz with 0.02 V amplitude (rms). A Metrohm Titrando pH meter (model 888) was used for pH measurements.

### 2.3. Preparation of MGPE/EGCG and MGPE/GT 

EGCG (4.5 mg) or GT (15 mg) and 150 mg graphite were thoroughly mixed together, following the addition of 5 mg paraffin oil. This mixture was ground using a mortar and pestle for 10 min. Next, the resulting paste was packed into a glass tube with a 2 mm diameter and a copper wire was inserted into the glass tube to act as the electrical contact. The new surface was obtained by pushing the carbon paste out of the glass tube and polishing with weighing paper. The electrodes were then placed in 0.1 M PBS at pH 2.0, and the electrode potential was cycled between −0.2 and 1.0 V (vs. Ag/AgCl) at a scan rate of 50 mV/s for 15 cycles in a cyclic voltammetry regime until a stable voltammogram was obtained. When not in use, the modified electrodes were stored in ultra-pure water.

### 2.4. Sample Preparation 

Samples of tablets containing AA (vitamin C, *n* = 5) were accurately weighed, powdered, dissolved in ultra-pure water, and then diluted with PBS (0.1 M, pH 2.0) to produce a solution of AA with a concentration of 100 mM. The electrochemical determination of AA in the tablet samples was performed as described above. The clinical laboratory human urine and blood serum samples were frozen at −20 °C immediately after collection and shipped. These samples were diluted five times with PBS prior to measurement. The standard addition technique was used for determining the AA, DA, and UA contents of the samples. Recovery of AA, DA, and UA in real samples was performed using the same procedure as described above.

## 3. Results and Discussion

### 3.1. Electrochemical Characterization of EGCG and MGPE/EGCG

**Figure 2:**
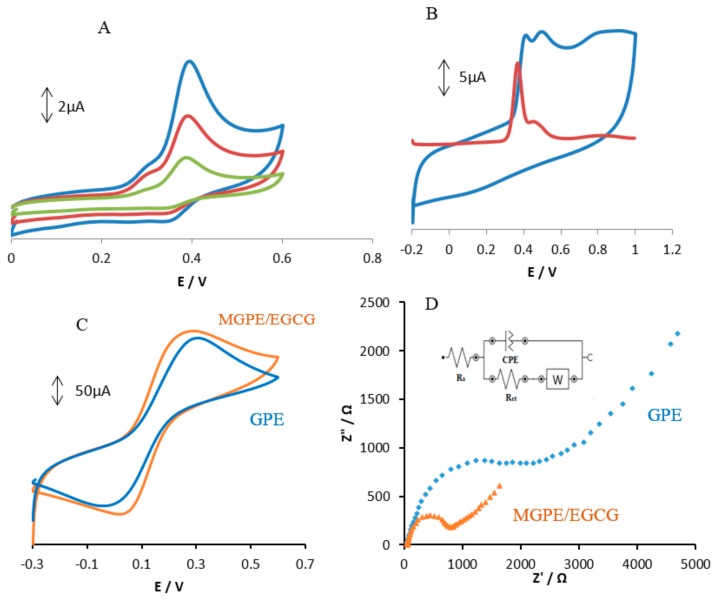
(**A**) CVs of EGCG at GCE at scan rates of 25, 50, and 100 mV/s; and (**B**) CV and DPV of a modified graphite paste electrode with epigallocatechin gallate (MGPE/EGCG) in PBS at pH 2 with a scan rate of 100 mV s^−1^; and (**D**) Nyquist plots of the GPE and MGPE/EGCG in 0.1 M KCl containing 10 mM Fe(CN)_6_^3−/4−^; (**D** inset) equivalent circuit. R_s_: solution resistance, R_ct_: charge transfer resistance, W: Warburg element. C_dl_: double layer capacitance. CVs of (**A**) the graphite paste electrode (GPE) and (**C**) modified graphite paste electrode (MGPE) in the presence of Fe(CN)_6_^3−/4−^ (1.0 mM) in 0.5 M KCl.

The cyclic voltammetry (CVs) and differential pulse voltammetry (DPV) of EGCG at glassy carbon electrode (GCE) are shown in Figure 2. Based on Figure 2A, there are two oxidation peaks at 0.29 and 0.38 V and two reduction peaks at 0.27 and 0.35 V for EGCG. The CV and DPV of MGPE/EGCG are shown in Figure 2B. Based on the DPV in this Figure, three peaks are shown: a peak at 0.36 V, a broad peak at 0.45 V, and a very broad and weak peak at 0.78 V. The CVs show three oxidation peaks at 0.40, 0.48, and 0.78 V. Figure 2C shows the CVs of GPE and MGPE/EGCG in 10 mM Fe(CN)_6_^3**−**/4**−**^ in 0.1 M KCl. The results indicate a decrease in the ∆E_p_ accompanied by an increase in the peak currents of the different electrodes as follows: MGPE/EGCG < GPE. 

Supporting evidence for these modified electrodes was found by EIS, a powerful technique to study electrode–electrolyte interfacial features. As shown in Figure 2D, the Nyquist plot of GPE comprises two parts: one semicircle—whose diameter equals the charge transfer resistance (R_ct_)—at higher frequencies, which indicates the charge transfer limitations, and a straight line that appears in low frequencies that signals the mass transfer limitations. The NOVA software was used for fitting and simulation of EIS data and also the Randles equivalent circuit illustrated in the inset of Figure 2D was selected as the equivalent circuit for fitting and simulation of EIS data. R_ct_, the active electrolyte resistance (R_s_), and the Warburg element (W) for GPE are 2.25 kΩ, 56.5 Ω, and 383 µΩ, respectively. The same elements for MGPE/EGCG are 663 Ω, 56.0 Ω, and 984 µΩ, respectively. Based on these results, the R_ct_ for MGPE/EGCG is 3.4 times less than for GPE. This indicates that the charge transfer resistance decreases to a lower value in the presence of EGCG for the MGPE/EGCG electrode.

**Figure 3:**
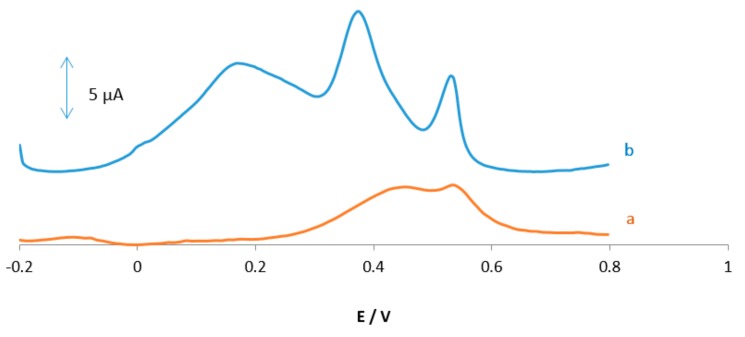
Differential pulse voltammograms of (**a**) GPE; and (**b**) MGPE/EGCG in 0.1 M PBS (pH 2.0) containing ascorbic acid (AA) (23 µM), dopamine (DA) (14 µM), and uric acid (UA) (14 µM).

The electrochemical behavior of the mixture of analytes containing AA, DA, and UA in 0.1 M PBS at pH 2.0 was carefully investigated at the surfaces of GPE and MGPE/EGCG using differential pulse voltammetry (DPV) and the results are shown in Figure 3. The GPE electrode shows two weak and broad oxidation peaks for DA and UA at 0.42 and 0.55 V, suggesting slow electron transfer kinetics and without any oxidation peak for AA (Figure 3a). In contrast, the MGPE/EGCG shows three well defined and sharp peaks for AA, DA, and UA at 0.17, 0.37, and 0.54 V, respectively. It can be seen from Figure 3 that the peak current for AA, DA, and UA at MGPE/EGCG is several times larger than that of the GPE due to the catalytic property of EGCG, wherein reduced EGCG acts as a mediator by increasing the rate of electron transfer. The potential peak separations for AA–DA and DA–UA are 0.21 V and 0.16 V, respectively, which is suitable for the simultaneous determination of three compounds. 

### 3.2. Effect of pH on the Oxidation of AA, DA, and UA 

**Figure 4:**
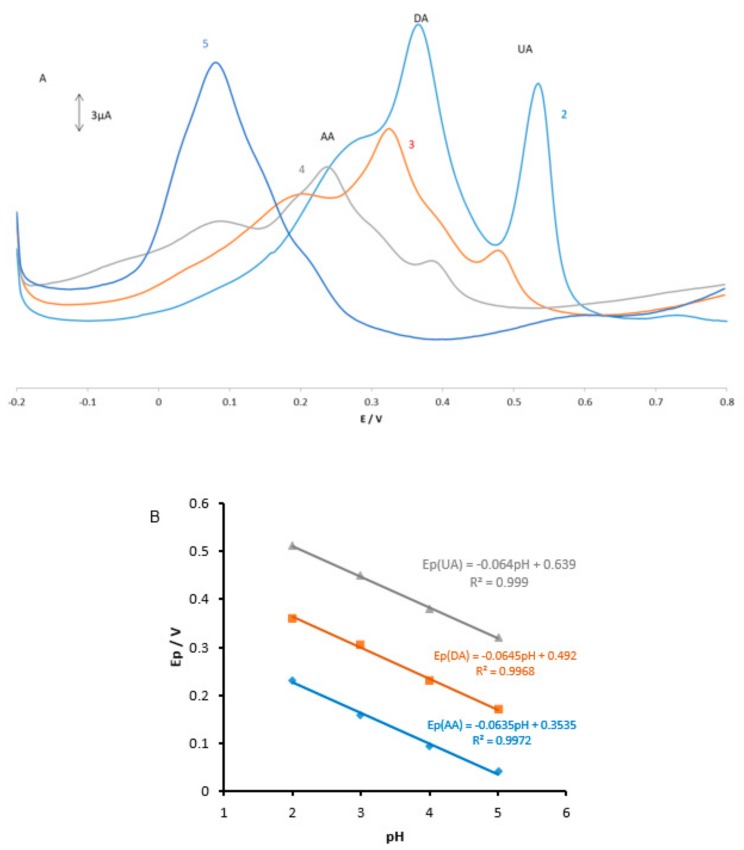
(**A**) Differential pulse voltammograms of MGPE/EGCG in 0.1 M PBS containing AA (9 µM), DA (4 µM), and UA (6 µM) at various pH (2–5); (**B**) plots of peak potential vs. pH for three analytes.

The pH effect at the MGPE/EGCG signal was carefully investigated by DPV in PBS solutions at pH levels ranging from 2 to 5. The results are shown in Figure 4. It is observed that as the pH of the medium is gradually increased, the peak potentials for the oxidation of AA, DA, and UA shift towards less positive values, showing that protons took part in the AA, DA, and UA electrooxidation reaction processes. A plot of Ep vs. pH for AA, DA, and UA in the working pH range is shown in Figure 4B. As can be seen, the Ep of all compounds has a linear relationship with buffer pH. The observed slopes of 0.0635, 0.0645, and 0.064 mV/pH for AA, DA, and UA, respectively, are close to the anticipated Nernstian value of 0.0585 V/pH for a two-electron, two-proton transfer in the electrochemical reaction. Therefore, it is suggested that the oxidation reaction of these analytes in the pH range of 2–5 involves two protons and two electrons (see Reactions 1–3). As shown in Figure 4A, PBS with pH 2 gives the best response in terms of peak current, peak shape; hence, it was selected as the optimal pH for further studies.


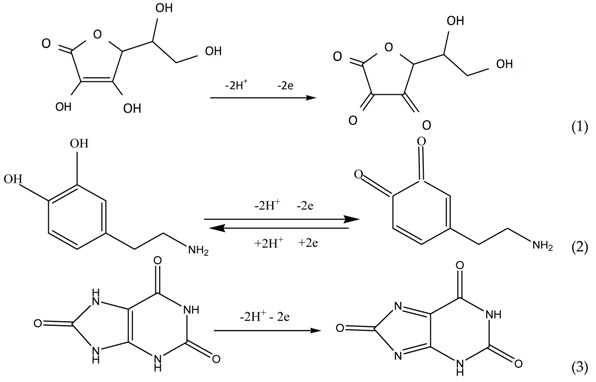


### 3.3. Chronoamperometric Studies

**Figure 5:**
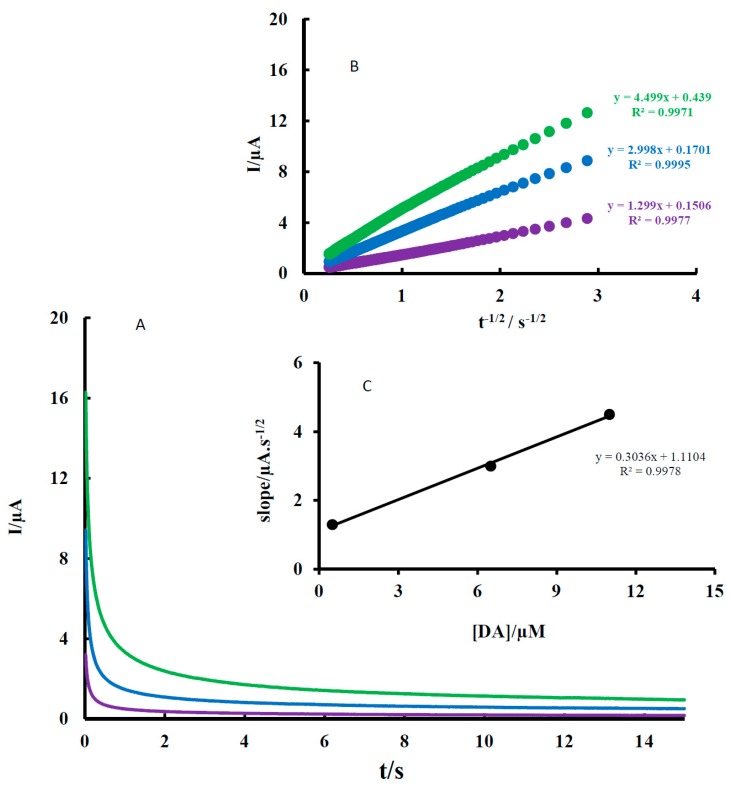
(**A**) Chronoamperograms of MGPE/EGCG for varying concentrations of DA: (1) 0.02, (2) 0.065, and (3) 0.11 mM in 0.1 M PBS (pH 2); (**B**) plots of anodic peak currents (I_pa_) vs. t^−1/2^; (**C**) plots of the slope of the straight line vs. DA concentration.

The diffusion coefficients of AA, DA, and UA were determined by a chronoamperometry method. A typical result for DA is shown in Figure 5. As shown, the diffusion coefficients of DA were determined by the Cottrell equation, which provides the variation of current with time for a diffusion-controlled process.

(1) $$I={\mathrm{nFACD}}^{1/2}\pi^{-1/2}t^{-1/2}$$

In this equation, D and C are the diffusion coefficients (cm^2^ s^−1^) and the concentration (mol cm^−3^), respectively. On the other hand, A and F are the surface area of the working electrode (0.0314 cm^2^) and the Faraday constant (96,485 C mol^−1^). Under diffusion control (mass transport), a plot of I vs. t^−1/2^ is linear and the value of D can be obtained from its slope. The value of D was found to be 1.47 × 10^−4^ cm^2^ s^−1^ for DA. The sample method was used for AA and UA and the value of D was determined to be 1.33 × 10^−6^ and 8.27 × 10^−5^ cm^2 −1^ for AA and UA, respectively. There are some reports for D of AA, DA, and UA in the literature. A comparison of these values is shown in Table 1. According to Table 1, the calculated D values in this report are within the acceptable range of the ones reported in the literature [20,21,22,23,24,25].

### 3.4. Interference Studies

**Figure 6:**
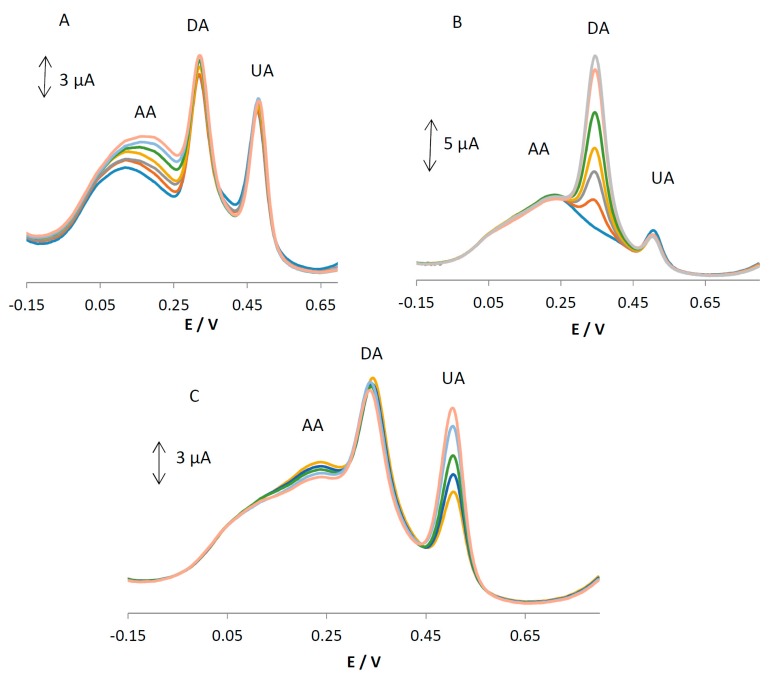
(**A**) Differential pulse voltammograms of MGPE/EGCG (**A**) containing DA (10 µM) and UA (13 µM) with varying concentrations of AA (15–23 µM); (**B**) containing AA (20 µM) and UA (7 µM) with varying concentrations of DA (2–14 µM); and (**C**) containing AA (23 µM) and DA (8 µM) with varying concentrations of UA (7–14 µM) in 0.1 M PBS (pH 2).

As AA, DA, and UA usually coexist in real samples, it is very important to study the interferences from each for the selective detection of one species. In each experiment, the concentration of one species was changed, while the concentrations of the other species were kept constant. The results are shown in Figure 6A–C. It can be seen from Figure 6A that the peak current of AA increases with an increase in the concentration of AA, while the peak current for the oxidation of DA and UA remains constant. As can be seen in Figure 6B,C, the voltammetric peaks corresponding to the oxidation of DA and UA increase linearly in accordance with an increase in their concentration of DA and UA, whereas the peak current for the oxidation of the other three compounds remains constant. The results show that the peak currents are linearly proportional to the concentration of AA (or DA and UA), while those of the other three analytes did not change; this indicates that the oxidation of AA, DA, and UA at MGPE/EGCG takes place independently.

### 3.5. Simultaneous Determination of AA, DA, and UA

**Figure 7:**
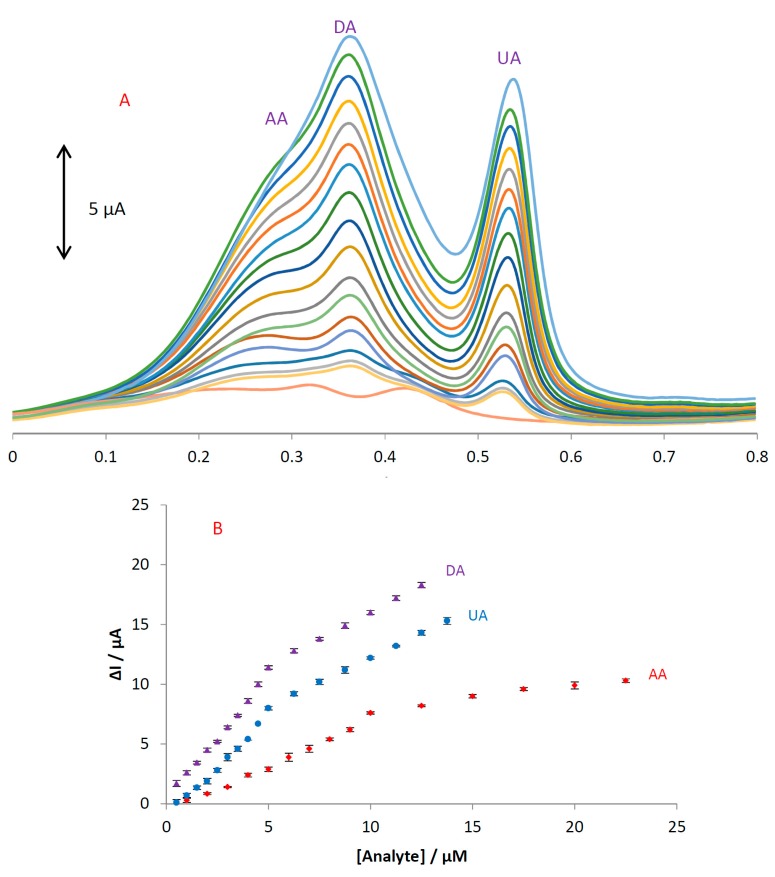
(**A**) Differential pulse voltammograms of MGPE/EGCG in 0.1 M PBS (pH 2) containing various concentrations of AA (1–23 μM), DA (0.5–14 μM), and UA (0.5–14 μM); (**B**) the plots of ∆Ip vs. concentration for AA, DA, and UA.

DPV was performed to investigate the relationship between the peak current and concentration of the three compounds. As shown in Figure 7, the DPV curves show three well-distinguished oxidation peaks. 

Electrocatalytic peak currents of AA, DA, and UA oxidation at the surface of MGPE/EGCG are linearly dependent on the analyte concentrations with two segments in the range of 1–10 and 10–23 μM for AA and 0.5–5 and 5–14 μM for both DA and UA. The regression equations for AA, DA, and UA were obtained as ΔI_p1_(AA) = 0.7924[AA] − 0.8033 (R^2^ = 0.9907), ΔI_p2_(AA) = 0.2194[AA] + 5.5343 (R^2^ = 0.9752), ΔI_p1_(DA) = 2.1178[DA] + 0.304 (R^2^ = 0.9918), ΔI_p2_(DA) = 0.8895[DA] + 7.1107 (R^2^ = 0.9986), ΔI_p1_(UA) = 1.7279[UA] − 1.2067 (R^2^ = 0.9849), and ΔI_p2_(UA) = 0.8248[UA] + 3.9679 (R^2^ = 0.9996), respectively. We hypothesized that the difference in the slopes for the calibration curves was due to different activities of the surfaces at low and high concentrations of the analytes. At lower concentrations of the analytes, due to a high number of active sites on the surface, the slope of the first calibration curve is high. While at higher concentrations of the analytes, due to decreasing active sites on the surface, the slope of the second segment of the calibration curve shows a decrease. The detection limit was calculated based on the relationship LOD = 3 S_blank_/m, where S_blank_ is the relative standard deviation of blank signals (*n* = 10) and m is the slope of the calibration plot. The theoretical detection limits were obtained as 190, 90, and 70 nM for AA, DA, and UA, respectively.

### 3.6. Real Sample Analysis

To evaluate the practical applicability of the proposed modified electrode, the MGPE/EGCG was utilized for the simultaneous determination of AA, DA, and UA in vitamin C tablets, human urine, and blood serum samples. The samples were diluted in PBS and DPV was performed for the simultaneous determination of AA, DA, and UA using the standard addition method. As depicted in Table 2, acceptable recovery values were obtained that indicate the applicability of MGPE/EGCG for trace amounts of these compounds in real sample analysis. 

### 3.7. Reproducibility, Repeatability, and Stability of the Sensor

**Figure 8:**
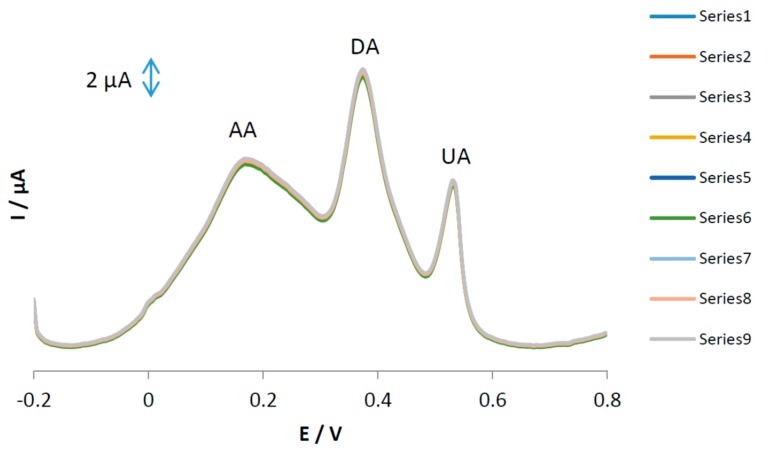
Differential pulse voltammograms of MGPE/EGCG in PBS at pH 2.0 for repetitive measurements (*n* = 9) of a solution containing of AA (23 µM), DA (14 µM), and UA (14 µM).

The stability and repeatability of the MGPE/EGCG surfaces were investigated using DPV for repetitive (*n* = 9) simultaneous determinations of AA, DA, and UA and the results are shown in Figure 8. Based on this figure, the relative standard deviations of the results were calculated as 1.25, 1.35, and 1.37 (%) for AA, DA, and UA, respectively. The reproducibility of the proposed electrode was studied using three different independent electrodes prepared with the same composition and the peak currents were recorded. The relative standard deviation of the sensor was calculated to be 2.89%. The long-term stability of the sensor was investigated by recording voltammograms of the modified sensor for a period of one month. During this time, the sensor response was recorded twice a week. The Ip_a_ values for AA, DA, and UA were retained at 95.3, 96.5, and 94.3%, respectively. Therefore, we conclude that the electrooxidation current responses of AA, DA, and UA at the MGPE/EGCG surface has excellent repeatability, reproducibility, and stability.

### 3.8. Simultaneous Determination of AA, DA, and UA Using MGPE/GT

**Figure 9:**
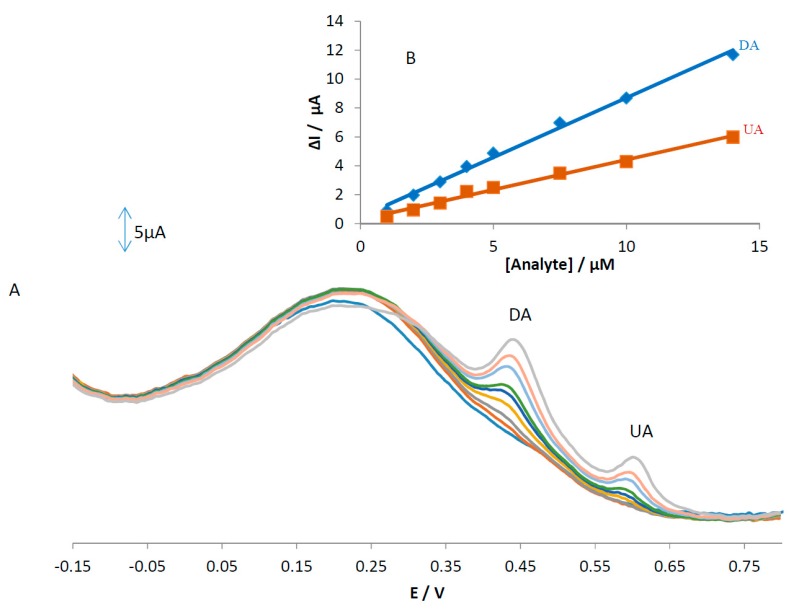
(**A**) Differential pulse voltammograms of MGPE/GT in 0.1 M PBS (pH 2) containing various concentrations of AA (1–25 μM), DA (0.5–14 μM), and UA (0.5–14 μM); (**B**) the plots of ∆Ip vs. concentration of DA and UA.

An electrode modified with green tea powder was used to simultaneously determine AA, DA, and UA and the results are shown in Figure 9. Based on this figure, only DA and UA show two oxidation peaks at 0.43 and 0.6 V without any oxidation peak for AA. The linear ranges are linear up to 14 µM with a detection limit of 0.18 and 0.33 µM for concentrations of DA and UA, respectively. The regression equations for DA and UA were obtained as ΔI_p_(DA) = 0.8243[DA] + 0.4585 (R^2^ = 0.9944) and ΔI_p_(UA) = 0.4152[UA] + 0.2639 (R^2^ = 0.9912), respectively. We hypothesized that the difference in the slopes for the calibration curves of MGPE/EGCG and MGPE/GT is due to the different activity of the modifier in the MGPE. The sensitivity values for determining DA and UA at the surface of the MGPE/EGCG are 2.6 and 4.2 (calibration curve slope ratio of the (MGPE/EGCG)/(MGPE/GT)), which is more than for MGPE/GT and is attributed to the modifier catalytic activity of the pure EGCG being greater than GT powder.

## 4. Conclusions

We have demonstrated for the first time a green tea derivative (EGCG) modified GPE for the simultaneous determination of AA, DA, and UA. We have also presented a GPE modified directly with green tea powder (Hamasaen, Japan) and have shown that we were able to successfully detect two out of the three analytes tested. Compared with the bare electrode, a significant increase in peak current was observed at the MGPE/EGCG electrode, which clearly demonstrates that EGCG can be used as an efficient modifier to enhance the kinetics of the electrochemical process of AA, DA, and UA. Optimization of the experimental conditions yielded a detection limit for AA, DA, and UA of 190, 90, and 70 nM, respectively. In addition, the presented sensor was successfully applied for the simultaneous determination of AA, DA, and UA in real samples with promising results for future development as a diagnostic device. A future application of this developed sensor could be as a wearable [26] or an ingestible electrochemical sensor [27]. Especially since the modifier (EGCG) used in this report is biocompatible, it is particularly attractive for future development as a wearable sensor for multiplexed electrochemical detection of clinically important analytes.

## Figures and Tables

**Table 1:** Calculated diffusion coefficients related to ascorbic acid (AA), dopamine (DA), and uric acid (UA) using a modified graphite paste electrode with epigallocatechin gallate (MGPE/EGCG) compared with the ones reported in the literature.

Analyte	D/cm^2^ s^−1^	Ref.
AA	1.99 × 10^−5^	[20]
	1.0 × 10^−6^	[21]
	5.77 × 10^−6^	[23]
	1.43 × 10^−4^	[24]
	9.35 × 10^−6^	[25]
	1.33 × 10^−6^	This work
DA	1.3 × 10^−5^	[23]
	7.9 × 10^−4^	[24]
	3.63 × 10^−5^	[25]
	1.47 × 10^−4^	This work
UA	1.50 × 10^−4^	[22]
	1.75 × 10^−5^	[23]
	2.8 × 10^−4^	[24]
	1.63 × 10^−4^	[25]
	8.27 × 10^−5^	This work

**Table 2:** Simultaneous determination of AA, DA, and UA in vitamin C tablets, human urine, and serum samples (*n* = 3). ‘Detected’ is the amount of analyte(s) present in the sample, ‘spiked’ is the amount of standard solution that was added to the sample, ‘found’ is the amount of spiked analyte concentration that was determined from the spiked solution.

Sample	Analytes		Detected (µM)	Spiked (µM)	Found (µM) (*n* = 3)	Recovery (%)
Vitamin C Tablet	I	AA	4	5	4.87	97.4
		DA	-	4	4.05	101.3
		UA	-	4	3.89	97.3
	II	AA	12	5	5.04	100.8
		DA	-	12	12.4	103.3
		UA	-	12	11.7	97.5
Urine	I	AA	-	5	4.80	96.0
		DA	-	5	4.91	98.2
		UA	4.5	5	5.06	101.2
	II	AA	-	5	4.75	95.0
		DA	-	5	4.87	97.4
		UA	7.4	5	5.15	103.0
Serum 1	AA		-	5	4.77	95.4
	DA		-	5	4.94	98.8
	UA		-	5	5.17	103.4
